# Selective Sensation Based Brain-Computer Interface via Mechanical Vibrotactile Stimulation

**DOI:** 10.1371/journal.pone.0064784

**Published:** 2013-06-06

**Authors:** Lin Yao, Jianjun Meng, Dingguo Zhang, Xinjun Sheng, Xiangyang Zhu

**Affiliations:** State Key Laboratory of Mechanical System and Vibration, Shanghai Jiao Tong University, Shanghai, China; University of Maryland, United States of America

## Abstract

In this work, mechanical vibrotactile stimulation was applied to subjects’ left and right wrist skins with equal intensity, and a selective sensation perception task was performed to achieve two types of selections similar to motor imagery Brain-Computer Interface. The proposed system was based on event-related desynchronization/synchronization (ERD/ERS), which had a correlation with processing of afferent inflow in human somatosensory system, and attentional effect which modulated the ERD/ERS. The experiments were carried out on nine subjects (without experience in selective sensation), and six of them showed a discrimination accuracy above 80%, three of them above 95%. Comparative experiments with motor imagery (with and without presence of stimulation) were also carried out, which further showed the feasibility of selective sensation as an alternative BCI task complementary to motor imagery. Specifically there was significant improvement (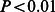) from near 65% in motor imagery (with and without presence of stimulation) to above 80% in selective sensation on some subjects. The proposed BCI modality might well cooperate with existing BCI modalities in the literature in enlarging the widespread usage of BCI system.

## Introduction

A brain-computer interface (BCI) provides a new non-muscular channel for communication and control with external world, which facilitates people who suffer from some sort of locked-in syndrome or amyotrophic lateral sclerosis [1]. A common way to gain BCI control is to use motor imagery of left and right hands, which is based on event-related desynchronization/synchronization(ERD/ERS) in specific frequency bands in the sensorimotor area of the brain [2]. ERD represents an attenuation of oscillation power in a given frequency band and mu ERD (7–12 Hz) is now generally accepted as representing cortical activation corresponding to active movement or motor imagery [3], [4]. While ERS corresponds to an increase in the amplitude, and beta ERS (13–25 Hz) has been interpreted as deactivation of cortical neurons involved in motor program execution or idle state of the motor system [5].

Brain oscillations in mu and beta bands have a strong correlation with contralateral movements [6]–[8] and sensory stimulation processing [9], [10]. Observation of significant beta ERS after electrical stimulation of finger and median nerve reveals a relationship between beta ERS and processing of afferent input [11], which has a bilateral distribution induced by unilateral stimulation. Meanwhile this beta ERS depends on the type and quantity of the afferent input. Many neuromagnetic imaging studies have shown these oscillatory activities within the human somatosensory cortex are strongly modulated by somatosensory stimulation and may reflect the normal processing of such stimuli [12]–[14]. Selective attention modulates somatosensory oscillations in alpha, beta, gamma bands that are both phase-locked and non-phase-locked to the stimulus [15], which results in significantly increased beta ERD/ERS, and alpha ERS due to attentional effects. Despite of attentional effect on ERD/ERS phenomena, steady-state somatosensory evoked potentials (SSSEPs) studies have revealed the attentional modulation of SSSEPs amplitude in humans, suggesting an enhancement of neural responses in the sense of flutter with attention [16].

Mueller-Putz [17] has established a SSSEPs based BCI, and attention modulation is the operating principle of such a system. In this a system, left and right index fingers are stimulated with different vibration frequencies, the classification accuracy ranged from 64% to 84% using lock-in amplifier features with linear discriminant analysis(LDA) as the classifier. Such a system has a big significance in development of BCI diversity, as there is a phenomenon called ‘BCI-illiteracy’ [18], different BCI modalities and combination of these, such as hybrid BCI [19], would promisingly improve the wide-spread uses of BCI system. In order to better cooperate with other BCI modalities, we propose an idea - Could selective sensation of mechanical vibrotactile stimulation of left and right wrist skins be used as a BCI modality? In such paradigm, left and right wrist skins are simultaneously stimulated with equal intensity and the same modulation frequency, and the subjects perform selective sensation of left and right afferent input. Revealed from the literature, ERD/ERS not just reflects motor programming but also has a strong correlation with somatosensory processing of afferent input, and ERD/ERS dynamics are modulated by attentional effects. Meanwhile lateral inhibition [14] and cortical gating [20] effect of simultaneously afferent inputs could make subjects feel that one stimulus is likely to be stronger than the other. These inspire us that the proposed paradigm could be possible. The focus of this work is to investigate whether selective sensation of vibrotactile stimulation of left and right wrist skins could be decoded on a single trial basis, and make a comparison with motor imagery of left and right hand. All the experimental conditions are the same except for the subjects’ mental tasks.

## Methods

### Subjects

Nine able-bodied subjects participated in these experiments, 6 males, 3 females, all right handed with mean age of 24 years old. Five of them had some BCI experience in motor imagery without presence of stimulation, but none of them had any experience in selective sensation task and motor imagery task with presence of stimulation. They were all informed with the whole experiment process. This study was approved by the Ethics Committee of Shanghai Jiao Tong University. All participants signed the informed consent forms before participating in the experiments.

### Stimulation Unit

In this experiment, stimulation was applied to the wrist skins, with 175 Hz (resonant frequency of the stimulating device) sinusoidal carrier frequency, modulated with 27 Hz to induce flutter sense. In this stimulating configuration, two types of mechanical receptor (Pacinian corpuscles and Meissner corpuscles) were stimulated, which were especially sensitive to frequency above 100 Hz and 20 to 50 Hz respectively [21]. And these stimulation configurations would attract much cortical processing of afferent inflow compared to single frequency stimulation either with high or low frequency, with which only one type of mechanical receptors was stimulated.

**Figure 1:**
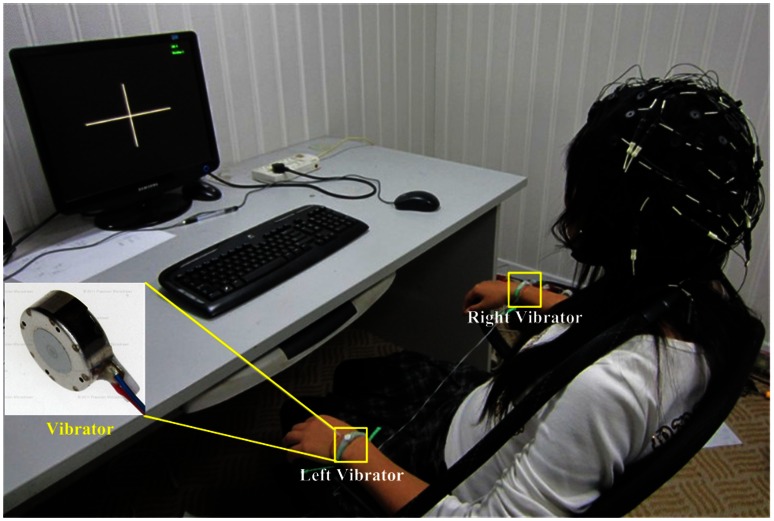
Experimental setup. The stimulation devices were attached to subject’s left and right wrist skins.

Both left and right wrist skins were simultaneously stimulated, as shown in Fig. 1, with equal amplitude and the same modulation frequency. The linear resonant actuators(10 mm, C10-100, Precision Microdrives Ltd. Typical Normalized Amplitude 1.4G) were used for vibrotactile stimulation. Electrical signal of 175 Hz sinusoidal carrier frequency modulated with 27 Hz sinusoidal frequency was produced via computer soundcard, and amplified with audio amplifier to drive the actuators. The amplitude of vibration was individually adjusted within the range of 0.5 times the device normalized amplitude to maximum amplitude of 11.3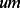 at resonant frequency, so that the subjects could feel the intense vibration with flutter sense, and it was modulated neither too small nor too strong that the subject could concentrate himself or herself on performing the predefined experimental task.

### EEG Recording

EEG signals were recorded using a SynAmps2 system(Neuroscan, U.S.A.). 64 channel quick-cap was used to collect 62 channel EEG signals, and the electrodes were placed according to the extended 10/20 system. The reference electrode was located on the vertex [22], and the ground electrode was located on forehead. An analog bandwidth filter with 0.5 Hz to 70 Hz and a notch filter with 50 Hz to diminish power line interference were applied to the original signals, which were sampled at 250 Hz.

### Experimental Paradigm

In the motor imagery task, the subjects were informed to mentally simulate kinaesthetic movement of their own left or right hand indicated from the cue, without any actual movements. And in the sensation task, the subjects were required to focus sensation on the indicated side of their hand as if stimulation on the attended side was stronger than the unattended side, while the stimulation applied at the both wrist skins was the same.

**Figure 2:**
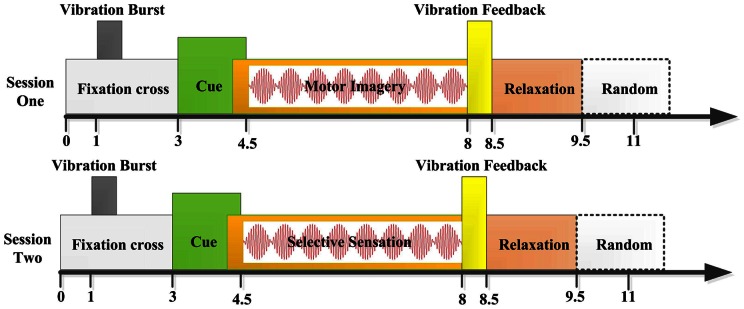
Trial structure of session 1 and session 2. The only difference between the two session was the subject’s task. One was motor imagery, the other was selective sensation.

During the EEG recording, the subjects sat in a comfortable armchair in the electrical shielded room. With both forearms and hands resting in the armrest, and the subjects should limit the eye blinks and stay still to avoid any facial or arm muscular movements. The subjects’ task was to perform motor imagery or selective sensation. In the first two sessions, the stimulation was applied to subjects’ wrist skin of both sides during the mental task. Every session contained four runs of 40 trials each, resulting 80 trials for each mental task. A total of 320 trials were performed by the subjects in the first two sessions, lasting for about an hour, and the subject got rested after each run. In the first session, the subject was required to perform motor imagery task of left and right hand, and during that time the vibrations were stimulated at the wrist skins. The procedure of single trial structure was given as follows. At the beginning of each trial, a fixation cross appeared in center of the screen. At the 1st second, a vibration burst with the same intensity stimulated both hands to attract the subject’s attention and be mentally ready for the subsequent task, with vibration time lasted for 200 ms. Then at the 3rd second, a red cue bar pointing either left or right was presented, which superimposed on the fixation cross and lasted for 1.5s. The subjects should perform the mental task after appearance of the cue bar. The mental task continued until to the 8th second, when the fixation cross disappeared. At the 4.5th second, the vibration applied to both hands with the same intensity, till to the end of the motor imagery. In the second session, the trial structure was the same, but the subjects’ task changed. At this session the subject performed selective sensation of left and right vibration afferent inflow according to the cue indication. During the first run of all the two sessions, there was no feedback. During all the subsequent three runs of each session, there would be vibration feedback, according to the on-line classification algorithm implemented within the experiment system. The feedback stimulus was applied according to the decoded task type either left or right, lasting for about 500 ms. After the feedback, there was a relaxation time period lasting for about 1s, during which the subjects should get relaxed and could blink his or her eyes. Then a random time period of about 0 to 2s was inserted after the relaxation period to further avoid subject’s adaptation, after that the next trial began. The whole trial structures were shown in Fig. 2.

Motor imagery with presence of stimulation in session one was mixed with cortical activity related to motor output and cortical processing of stimulation induced afferent inflow. Aiming to better understand what components came from the sensation task and what from the motor imagery task, all the subjects took one additional session. In the session three, the trial structure was the same as in the session one and two, except no stimulation was applied to the wrist skins during the motor imagery period(motor imagery without presence of stimulation). The subjects were required to have enough rest period of about 15 minutes between two sessions.

### Algorithms and Adaptation Strategy

Decoding algorithm for both motor imagery task and sensation task is mainly based on Common Spatial Pattern (CSP), which is widely used in motor imagery based BCI literature. Mathematically it is realized by simultaneous diagonalization of the covariance matrices for the two classes [23], [24]. The raw EEG signals are represented as 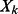 with dimensions 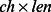, where 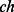 is the number of recording electrodes, and 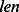 is the number of sample points. After preprocessing time segmentation and band pass filtering within the certain frequency band, the normalized spatial covariance of the EEG can be obtained from
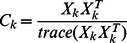
(1)where 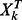 denotes the transpose of the matrix 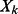, and 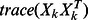 is the sum of the diagonal elements of the matrix 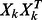. Let
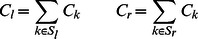
(2)where 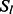 and 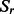 are the two index sets of the separate classes. Denote the composite spatial covariance 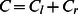, and 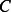 is decomposed as
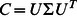
(3)where 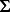 is the diagonal matrix of the eigenvalues and 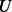 is the matrix of the corresponding eigenvectors. Using whitening transformation



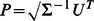
(4)the spatial covariances of 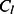 and 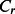 can be transformed as.
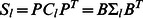
(5)

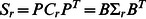
(6)


where 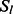 and 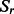 share common eigenvectors 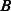, and.
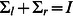
(7)



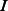
 is the identity matrix. Denote the projection matrix 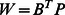 is of dimension 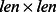. The rows of 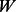 are called spatial filters, and the columns of 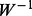 are called spatial patterns. To the 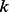-th trial, the filtered signal 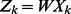 is uncorrelated. In this work, the log variance of the first three rows and last three rows of 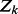 corresponding to the largest three eigenvalues and the smallest three eigenvalues are chosen as feature vectors.

**Figure 3:**
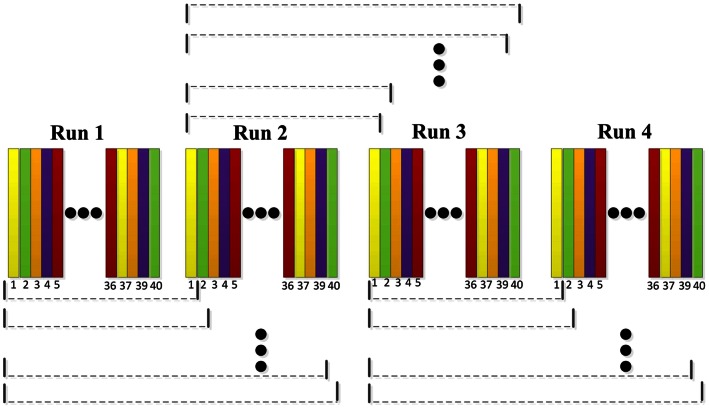
Adaptation strategy used in the on-line experiment. Each run contained 40 trials, the classification of current trial was based on 40 trials in the previous run and trials before the current trial in the same run.

In order to better motivate the subjects that in anticipating the experiment, feedback was important so that the subject could actively interact with the BCI system and concentrate more on the performing task. Also the adaptation was necessary to better pursuit the state changes of the brain. In this experiment design, the label of the EEG segment was known from the paradigm, and was used to retrain spatial filters and LDA classifier. In the first run, no feedback was presented, and the 40 trials within the first run were used to train spatial filters via CSP algorithm and establish LDA classifier [25], [26]. From the 2nd run to the 4th run, the feedback was presented based on the trials before the current trial in the same run and trials in the previous run, and new spatial filter and LDA classifier were re-trained after each trial, which was used to classify the upcoming trial, as shown in Fig. 3.

## Results

### Off-line and On-line Analysis of Selective Sensation Session

**Figure 4:**
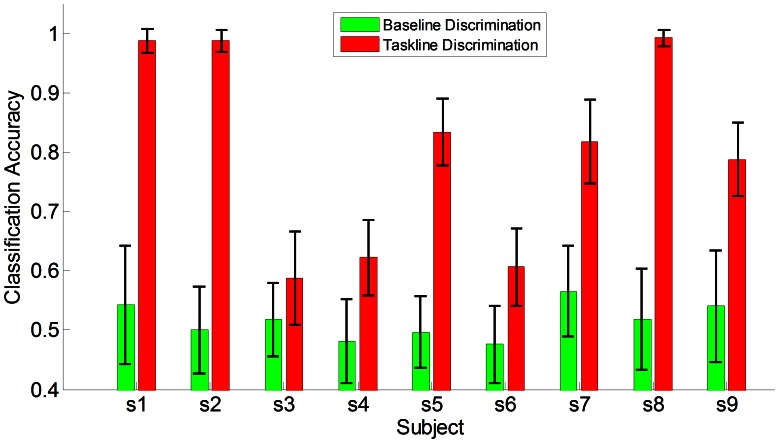
Comparison of baseline discrimination and taskline discrimination in sensation session. Baseline indicated the time from the 1st second to the 3rd second from the start of the trial(2 to 0 second before stimulus of the cue), Taskline indicated the time interval from the 4th second to the 7th second from the start of the trial(1 to 4 second post stimulus of the cue).

**Figure 5:**
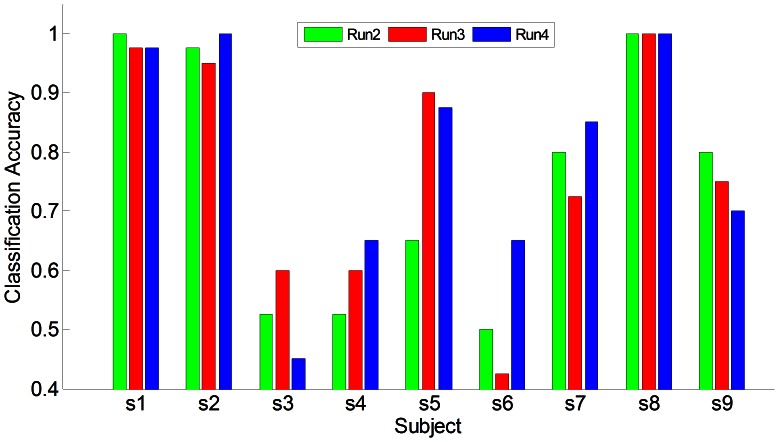
On-line classification accuracy from nine subjects in the sensation session.

The time interval for on-line and off-line analysis of discrimination between left and right motor imagery or left and right stimulation selective sensation was chosen from the 4th second to the 7th second at the beginning of the trial(1 to 4 second post to the stimulus of the cue), and the frequency band was chosen to cover the alpha and beta band of 8 Hz to 26 Hz, using 4th-order butterworth filter. The processing procedure was the same for both motor imagery task and sensation task. A 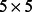 fold cross validation was adopted to evaluate the classification accuracy between left and right. It worked as follows: first randomly permutated the trials gathered in one session(160 left and right trials in our experiments), then equally divided into five partitions, every partition was used as an unknown test set which was classified by the classifier trained using the remaining four partitions, resulting a classification accuracy for each of the partition. Finally this process was repeated five times, generating 25 classification accuracies for statistical evaluation of the discrimination of the mental tasks. In convince of the discrimination between left and right selective sensation, baseline EEG of 2s before the indicating cue was extracted and evaluated in the same procedure, as shown in Fig. 4. Baseline activity showed no discriminative information of random chance level between the two classes, while taskline activity involved with subject’s mental task exhibited significant discrimination difference from the baseline with 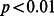. Clearly, during the base-line period the subject performed nothing but waited for the indicating cue, so the EEG signals showed plain discriminative information. During the taskline period, six of them achieved a classification accuracy above 80%, and three of the six above 95%, while other three showed less discriminative information of about 60%. Fig. 5 showed the on-line classification of each subject across the run 2 to run 4(run 1 was used for establishing the classifier), and subjects s1, s2, s8 showed a stable performance.

### Motor Imagery with and without Presence of Stimulation

**Figure 6:**
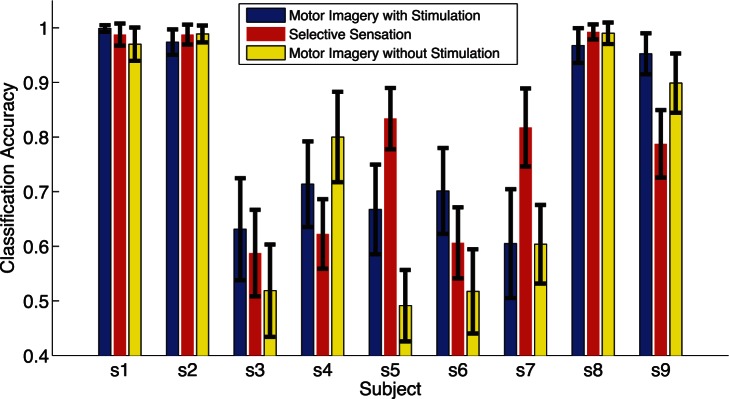
Comparision of discrimination accuracy of left and right among motor imagery with and without presence of stimulation, and selective sensation. The blue bars indicated the discrimination accuracy of left and right hand motor imagery with presence of stimulation in session one. The red bars indicated the discrimination accuracy of left and right selective sensation in session two. The yellow bars indicated the discrimination accuracy of left and right hand motor imagery without presence of stimulation in session three.

In order to better understand the task difference between Motor Imagery with and without presence of stimulation and Selective Sensation, left and right classifications of session one(motor imagery with presence of stimulation), session two(selective sensation), session three(motor imagery without presence of stimulation), were compared as shown in Fig. 6. Subjects s1, s2, s8 showed classification accuracy of above 95% in all the three sessions, while subject s5 and s7 showed an improved classification accuracy of above 80% for reliable control in selective sensation BCI, which was significant improvement from motor imagery with and without presence of stimulation(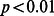) and might be especially useful for people who were in difficulty with motor imagery BCI. While there was a drop in subject s9 from 90% accuracy in motor imagery in session one and three to 80% in selective sensation in session two. Subject s3, s6 hardly reached classification accuracy of 70% in almost all the three sessions, and subject s4 showed ability in control using motor imagery without presence of stimulation. In all, the results showed selective sensation task could be used as an additional BCI modality, and motor imagery with presence of stimulation could still be decoded as motor imagery without presence of stimulation in the literature.

### Optimal Selection of Time Segment and Frequency Band

**Figure 7:**
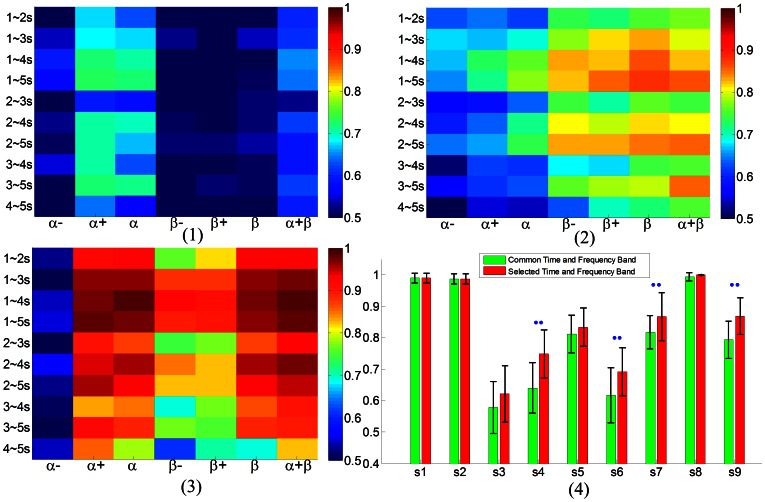
Discriminative accuracy under different combinations of time segmentation and frequency band, and selection of optimal time segment and frequency band in sensation session. (**1**) Discriminative information under different time segmentations and frequency bands from subject s4. The most discriminative information was concentrated on alpha band and showed little discriminative information in the entire beta band. The color bar indicated the classification accuracy. (**2**) Discriminative information from subject s7. The most discriminative information was concentrated on beta band and showed less discriminative information in alpha band. (**3**) Discriminative information from subject s2. The discriminative information was on both alpha and beta band, but lower alpha band showed little discriminative information. (**4**) Improvement after optimal selection of time and frequency band for each individual. Common time and frequency band corresponded to [1 4]s post appearance of the indicating cue and [8 26]Hz maintaining both alpha and beta rhythm. The selected time and frequency band were chosen such that the subject could achieve the best discrimination performance. Two point markers indicated significant improvement using *T-test* with 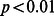.

To get a full understanding of the role of different time segments and frequency bands in the classification of the left and right sensation task, time segments and frequency band divisions were described as follows. In the frequency domain, lower alpha 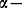 [8 10]Hz, upper alpha 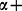 [10 13]Hz, alpha 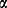 [8 13]Hz, and lower beta 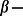 [13 20]Hz, upper beta 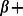 [20 26]Hz, and beta 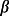 [13 26]Hz, and both alpha and beta band 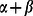 [8 26]Hz were divided. In the time domain, as reaction time (from the appearance of the indicating cue to the actual mental performing) of each subject existed, and varied from subject to subject, so we focused on the time 1s after appearance of the cue to 5s. The time divisions were as 1
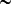
2 s, 1
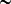
3 s, 1
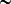
4 s, 1
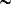
5 s, and 2
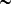
3 s, 2
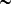
4 s, 2
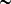
5 s, and 3
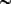
4 s , 3
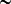
5 s, and 4
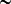
5 s. Fig. 7(1)(2)(3), exhibited different distribution of discrimination information across three subjects, which showed different dominant rhythmical bands in classifying the left and right sensation. After proper selection of time and frequency band to each individual, an obvious improvement was shown in Fig. 7(4), especially for subjects s4, s6, s7, s9 with 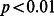.

## Discussion

Six out of nine subject’s performance were above 80%, and three of them above 95%, which proved the efficacy of the proposed selective sensation based BCI system. This system might be especially useful for those who lost their volitional eye control, while their somatosensory systems remained able to work, such as patients suffering from some sort of locked-in syndrome, or in late stages of amyotrophic lateral sclerosis. The visual evoked potential (VEP) based BCI, such as P300 component after the stimulus or steady-state visual evoked potential (SSVEP), which had a much higher information transfer rate(ITR) would be limited in such a group of patients. Meanwhile, when using P300 or SSVEP based BCI, subject’s eyes must be concentrated on the visual stimuli (steady flashing with certain frequency or oddball based random flashing), inadequately the subject could not engage in other everyday normal activities. With the use of selective sensation, the subject’s eyes could anticipate in other normal activities. Still, selective sensation could easily cooperate with other BCI modality systems to develop multi-modal BCI or hybrid BCI, which can be better adaptive to different BCI users and enhance the ranges of BCI usage. As from the study proposed by Allison et al. [27], motor imagery and SSVEP based hybrid BCI would be used for people who couldn’t attain effective communication via conventional BCI. Meanwhile by better integration, improved classification accuracy, reduced selection time and increasing number of possible commands could be achieved, which in turn can enlarge the number of BCI users.

**Figure 8:**
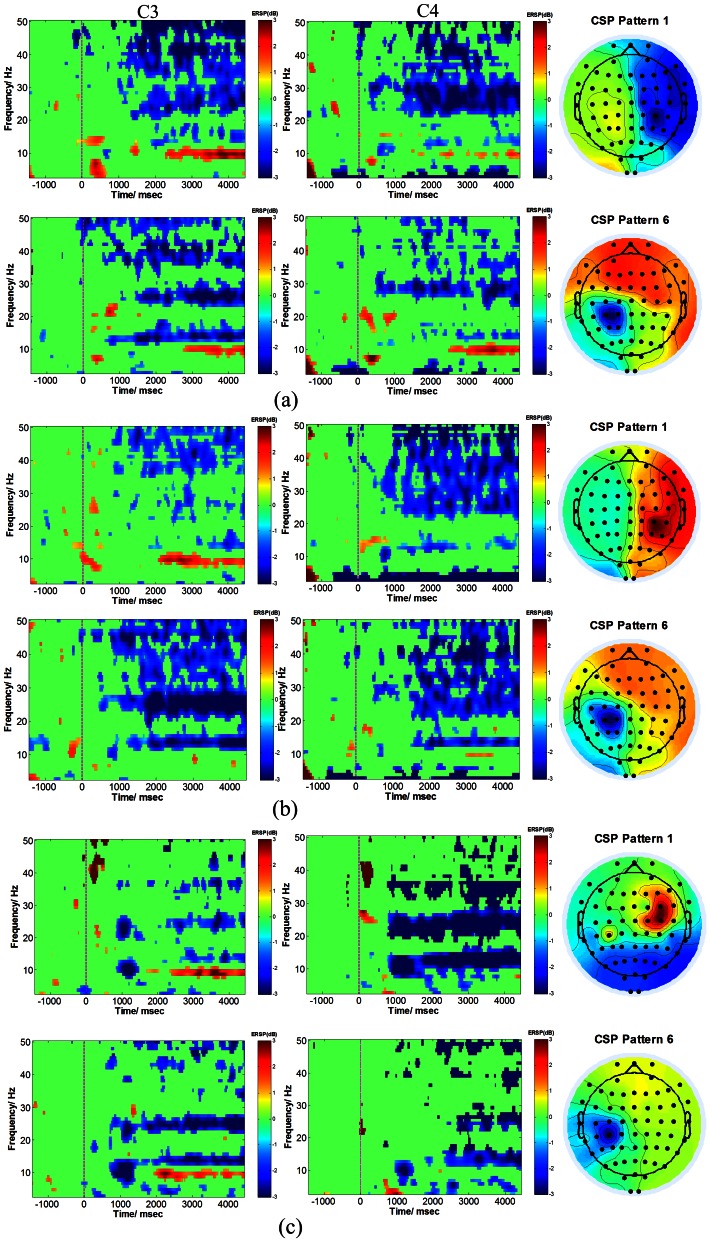
Event Related Spectrum Perturbation(with bootstrap significance *p* = 0.01), and topograph of CSP pattern from subject 1 in separate three sessions. (**a**) Motor Imagery with presence of stimulation, upper row corresponded to left class while lower row corresponded to right class both in C3 and C4 channels referenced to vertex of the brain. (**b**) Selective sensation. (**c**) Motor Imagery without presence of stimulation.

Further, event related spectrum perturbation(ERSP) at the critical channels of C3 and C4 [13], [28], and topograph of CSP patterns 1 and 6 corresponding to the largest and smallest eigenvalues from subject 1 during three separate sessions, were compared as shown in Fig. 8. It was calculated every 200 ms with hanning tapper, convoluted with modified sinusoid basis in which the number of cycles linearly changed with frequency [29] to achieve proper time and frequency resolution, and nonsignificant parts were wiped out under bootstrap significance level of 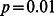 [30], using fieldtrip toolbox [31] and eeglab [32]. All three sessions exhibited much similar CSP patterns, which showed the effectiveness in discrimination of left and right selective sensation like motor imagery. Motor imagery without presence of stimulation in session three represented as the pure cortical motor efferent process, and stimulation sensation in session two represented as pure cortical processing of afferent inflow, while Motor Imagery with presence of stimulation in session one represented as the mixture of motor efferent outflow and stimuli induced afferent inflow. From the significance plot of ERSP in Fig. 8, a wider ERD distribution in the frequency domain is shown in session one and two compared to session three, mainly because of the stimulation applied in the session one and two. Interestingly, we found in right motor imagery with and without presence of stimulation there was an significant ERS around 10 Hz in C3(contralateral to the right hand), while in right selective sensation there wasn’t any significant ERS around 10 Hz, which might contribute to the mental task difference.

From the time/frequency segmentation and jointly combination, vibrotactile sensation of left and right exhibited different discrimination accuracy distributions. As can be seen from subject s4, the well discriminative frequency band lay in the upper alpha band, while in subject s7, the well discriminative frequency band lay in beta band. In contrast, for subject s2 the discriminative frequency band lay in both upper alpha and beta bands. From the time segmentation point of view, the longer the time, the better the classification accuracy could be achieved. After optimal selection of time and frequency bands, the average classification achieved is 3.43% higher than the common time and frequency band. Interestingly, lower alpha band showed less discriminative information in distinguishing of left and right wrist vibrotactile sensation, while upper alpha band showed more distinguishable information. It was in consistence with that in [33]. There was a functional dissociation of lower and upper frequency mu rhythms, the lower frequency component resulted in a widespread movement-type non-specific ERD pattern, whereas the upper frequency component showed a more focused and movement-type specific pattern. In comparison, to sensory afferent input selective sensation modality, there might also exist sensation non-specific and sensation specific rhythm, which were shown in the discrimination information distribution in the various time segments and frequency bands.

Study [34] has shown that amplitudes of the event related potential(ERP) or field(ERF) components following simultaneous stimulation are in general smaller than the arithmetic sum of the amplitudes for separate stimulation, this violation against the linear addition effect of linear system was known as sensory gating. Sensory gating effect might contribute to the discrimination of left and right selective sensation, and two neuronal mechanisms cortical gating and active lateral inhibition might well explain the sensory gating effect. Simultaneously stimulating remote left and right resulted in less lateral inhibition effect than simultaneously stimulating of the adjacent fingers of the same hand, because of somatotopically organized sensorimotor cortex [14]. Attentional modulation of cortical sensory gating effect presumably played an important role in the selective processing of afferent input, as revealed from everyday experience that perception of the attended afferent input is likely to be stronger than others although they are simultaneously applied with equal intensity.
